# A Survival Tree of Advanced Melanoma Patients with Brain Metastases Treated with Immune Checkpoint Inhibitors

**DOI:** 10.3390/cancers15112922

**Published:** 2023-05-26

**Authors:** Olivier J. van Not, Thijs T. Wind, Rawa K. Ismail, Arkajyoti Bhattacharya, Mathilde Jalving, Christian U. Blank, Maureen J. B. Aarts, Franchette W. P. J. van den Berkmortel, Marye J. Boers-Sonderen, Alfonsus J. M. van den Eertwegh, Jan Willem B. de Groot, John B. Haanen, Ellen Kapiteijn, Manja Bloem, Djura Piersma, Rozemarijn S. van Rijn, Marion Stevense-den Boer, Astrid A. M. van der Veldt, Gerard Vreugdenhil, Michel W. J. M. Wouters, Willeke A. M. Blokx, Karijn P. M. Suijkerbuijk, Rudolf S. N. Fehrmann, Geke A. P. Hospers

**Affiliations:** 1 Scientific Bureau, Dutch Institute for Clinical Auditing, Rijnsburgerweg 10, 2333 AA Leiden, The Netherlands; 2 Department of Medical Oncology, University Medical Centre Utrecht, Heidelberglaan 100, 3584 CX Utrecht, The Netherlands; 3 Department of Medical Oncology, University Medical Centre Groningen, University of Groningen, Hanzeplein 1, 9713GZ Groningen, The Netherlands; 4 Department of Molecular Oncology & Immunology, Netherlands Cancer Institute, Plesmanlaan 121, 1066 CX Amsterdam, The Netherlands; 5 Department of Medical Oncology & Immunology, Netherlands Cancer Institute, Plesmanlaan 121, 1066 CX Amsterdam, The Netherlands; 6 Department of Medical Oncology, GROW-School for Oncology and Reproduction, Maastricht University Medical Centre+, P. Debyelaan 25, 6229 HX Maastricht, The Netherlands; 7 Department of Medical Oncology, Zuyderland Medical Centre Sittard, Dr. H. van der Hoffplein 1, 6162 BG Sittard-Geleen, The Netherlands; 8 Department of Medical Oncology, Radboud University Medical Centre, Geert Grooteplein Zuid 10, 6525 GA Nijmegen, The Netherlands; 9 Department of Medical Oncology, Cancer Center Amsterdam, VU University Medical Center, Amsterdam UMC, De Boelelaan 1118, 1081 HZ Amsterdam, The Netherlands; 10 Department of Medical Oncology, Isala Oncology Center, Dokter van Heesweg 2, 8025 AB Zwolle, The Netherlands; 11 Department of Medical Oncology, Leiden University Medical Centre, Albinusdreef 2, 2333 ZA Leiden, The Netherlands; 12 Department of Biomedical Data Sciences, Leiden University Medical Centre, Einthovenweg 20, 2333 ZC Leiden, The Netherlands; 13 Department of Surgical Oncology, Netherlands Cancer Institute, Plesmanlaan 121, 1066 CX Amsterdam, The Netherlands; 14 Department of Internal Medicine, Medisch Spectrum Twente, Koningsplein 1, 7512 KZ Enschede, The Netherlands; 15 Department of Internal Medicine, Medical Centre Leeuwarden, Henri Dunantweg 2, 8934 AD Leeuwarden, The Netherlands; 16 Department of Internal Medicine, Amphia Hospital, Molengracht 21, 4818 CK Breda, The Netherlands; 17 Department of Medical Oncology and Radiology & Nuclear Medicine, Erasmus Medical Centre, ‘s-Gravendijkwal 230, 3015 CE Rotterdam, The Netherlands; 18 Department of Internal Medicine, Maxima Medical Centre, De Run 4600, 5504 DB Eindhoven, The Netherlands; 19 Department of Pathology, University Medical Centre Utrecht, Heidelberglaan 100, 3584 CX Utrecht, The Netherlands

**Keywords:** advanced melanoma, immunotherapy, brain metastases, survival tree

## Abstract

**Simple Summary:**

Up to 50% of patients diagnosed with advanced melanoma develop brain metastases during the course of their disease. The prognosis of melanoma patients is heavily affected by the presence of brain metastases. Unfortunately, there is a lack of data on prognostic factors for these patients. Many of these patients are treated with immune checkpoint inhibitors. Therefore, the aim of our study was to identify prognostic factors in melanoma patients with brain metastases treated with immune checkpoint inhibitors. In a population of 1278 advanced melanoma patients, we found that serum lactate dehydrogenase levels were the strongest clinical parameter associated with survival. This information is useful for both doctors and patients to provide more insight into patients’ prognoses.

**Abstract:**

The efficacy of immune checkpoint inhibitors (ICIs) in patients with advanced melanoma that develop brain metastases (BM) remains unpredictable. In this study, we aimed to identify prognostic factors in patients with melanoma BM who are treated with ICIs. Data from advanced melanoma patients with BM treated with ICIs in any line between 2013 and 2020 were obtained from the Dutch Melanoma Treatment Registry. Patients were included from the time of the treatment of BM with ICIs. Survival tree analysis was performed with clinicopathological parameters as potential classifiers and overall survival (OS) as the response variable. In total, 1278 patients were included. Most patients were treated with ipilimumab–nivolumab combination therapy (45%). The survival tree analysis resulted in 31 subgroups. The median OS ranged from 2.7 months to 35.7 months. The strongest clinical parameter associated with survival in advanced melanoma patients with BM was the serum lactate dehydrogenase (LDH) level. Patients with elevated LDH levels and symptomatic BM had the worst prognosis. The clinicopathological classifiers identified in this study can contribute to optimizing clinical studies and can aid doctors in giving an indication of the patients’ survival based on their baseline and disease characteristics.

## 1. Introduction

The introduction of targeted therapies and immunotherapies in advanced melanoma care has significantly improved the prognosis of these patients [1,2,3]. Up to 50% of the patients diagnosed with advanced melanoma develop brain metastases (BM) during the course of their disease. Melanoma is one of the most common cancer types in which the tumor spreads to the brain [4,5,6,7]. Furthermore, after lung and breast cancer, melanoma is the third most common diagnosis in patients with BM [8]. Treatment options for melanoma BM used to be limited, consisting of chemotherapy, whole brain radiotherapy, or surgery. Now, besides surgery and radiotherapy, immunotherapy treatment and targeted therapy are the cornerstones of managing BM. The immunotherapies used in daily clinical practice are ipilimumab, which is less frequently administered now, pembrolizumab, nivolumab, and a combination therapy of ipilimumab and nivolumab. Phase III clinical trials investigating new systemic therapies often exclude patients with BM, especially when symptomatic [9,10,11]. Previous Dutch research has shown that 40% of the melanoma population is considered ineligible for trial participation [12]. Nearly 70% of ineligible patients had BM, and BM are a significant part of the treatment landscape. Research has shown the negative impact of BM on the survival of advanced melanoma patients [13]. Despite this negative impact, the prognosis of patients with melanoma BM diagnosed between 2015 and 2019 improved compared to those diagnosed between 2010 and 2014, with median overall survival (OS) increasing from 9 months to 13 months. One of the factors associated with the improvement in survival was immunotherapy [14].

Since advanced melanoma patients with BM were mostly excluded from the phase III clinical trials investigating immunotherapy, information about the effectiveness of immune checkpoint inhibitors (ICIs) in patients with BM is limited. Phase II/III trials that did include this patient group suggest a clinical benefit of immune checkpoint inhibitors for melanoma patients with BM [15,16]. In these studies, some trial patients had a durable response to ICIs. However, for symptomatic patients, the chances of obtaining a durable response are limited. The number of included patients in these studies were relatively small, with a low proportion of patients with symptomatic BM. As a consequence, there is a lack of data on prognostic survival factors such as performance status and serum lactate dehydrogenase (LDH) levels in patients with BM. In this study, we aimed to identify prognostic survival factors in a set of advanced melanoma patients with BM treated with ICIs, using survival tree analysis to evaluate the clinical parameters associated with survival.

## 2. Methods

### 2.1. Patients

The study population comprised all patients with melanoma BM (symptomatic or asymptomatic) who were treated with immune checkpoint inhibitors in any treatment line in the Netherlands between 2013 and 2020. A treatment line was defined as systemic treatment after the diagnosis of advanced melanoma. For each patient, the first systemic treatment line of ICIs after a diagnosis of BM was used for the analysis. The information used as input for the survival tree analysis was derived from this specific treatment line. Patients with uveal melanoma or who were under the age of 18 were excluded.

### 2.2. Data Source

The data were derived from the Dutch Melanoma Treatment Registry (DMTR), which is a prospective population-based registry including all Dutch advanced melanoma patients and was initiated in 2013 [17]. All fourteen Dutch melanoma centers register their patients in the DMTR. Data registry is performed by trained data managers. The DMTR consists of >700 registry items, including patient and tumor characteristics, treatment details, and clinical outcomes. This research was not deemed subject to the Medical Research Involving Human Subjects Act, in compliance with Dutch regulations.

### 2.3. Statistical Analysis

Descriptive statistics were used to summarize patient and tumor characteristics. Kaplan–Meier estimates were used to calculate the study population’s OS probabilities. Survival times were calculated from the start of ICI for the treatment of BM until death or last follow-up. The median follow-up time was estimated with the reverse Kaplan–Meier method. Comparisons were considered statistically significant for 2-sided *p*-values < 0.05. Data handling and statistical analyses were performed using RStudio (version 4.0.2).

### 2.4. Survival Tree Analysis

Survival tree analysis was performed using clinicopathological variables as potential classifiers. Patients with missing clinicopathological variables were excluded from the survival tree analysis. The clinicopathologic parameters used were the following: age at diagnosis, gender, stage according to the eighth edition of the American Joint Committee on Cancer (AJCC) [18] at diagnosis, type of BM (symptomatic or asymptomatic) at first systemic treatment line at which the patient receives ICIs, location of the primary melanoma tumor, type of melanoma, number of organ sites with metastases, presence of liver metastases, ECOG PS, LDH level, presence of *BRAF* mutation, presence of *NRAS* mutation, type of first ICIs for BM, line of systemic treatment in which ICIs were first given (of which the count started at the first systemic treatment line after the diagnosis of the advanced melanoma), prior surgery, surgery of the brain, prior radiotherapy, radiotherapy of the brain, type of radiotherapy, BRAF therapy prior to BM diagnoses, BRAF therapy after diagnoses of BM, ipilimumab therapy prior to BM diagnoses, and anti-PD-1 therapy prior to BM diagnoses. Patients with missing values in the above-mentioned variables and missing values in the time to the event were excluded from the survival tree analysis. Additional information regarding the survival tree analysis can be found in the (Supplementary Methods). For each classifier, two subsets of patients were obtained at each possible cut-off, and survival probabilities were compared using the log-rank statistic. Next, patients were divided into subsets based on the most significant classifier at the optimal cut-off. This process was repeated recursively on the resulting subsets. The recursion stopped if the combined number of patients in the 2 subsets dropped to <100, the number of uncensored events in both subsets combined was <50, or the number of patients in 1 of the subsets was <34. Classifier robustness was assessed using 10,000 iterations, with random selections of 80% of the patients. Robustness was evaluated by correlating the classifier ranks based on their significance in each iteration with the classifier ranks in the original survival tree.

## 3. Results

### 3.1. Patient Characteristics

From 2013 to 2020, 6819 advanced melanoma patients were registered in the DMTR. Of these patients, 2362 developed BM. Of these 2362 patients, we excluded 7 patients with BM and uveal melanoma, and 1072 were excluded because they did not receive ICI treatment after the diagnosis of their BM. This resulted in 1278 patients that met the inclusion criteria for this study. Median follow-up was 30.4 months (95%CI 28.1–33.6). Of the 1278 patients, 229 had 1 or more clinicopathological variables missing, and 27 had missing information regarding survival outcomes. This resulted in 1022 patients being included in the survival tree analysis. Summaries of patient and tumor characteristics at the time of the treatment of the melanoma BM with ICIs can be found in (Table 1). To the 1278 patients, ipilimumab–nivolumab was the most frequently administered treatment (*n* = 571; 45%), followed by anti-PD-1 (*n* = 481; 38% of which *n* = 204; 16% nivolumab and *n* = 277; 22% pembrolizumab), and ipilimumab monotherapy (*n* = 226; 18%). Of the 1278 patients, 977 patients had BM at their first diagnosis of advanced melanoma, of which 37% had asymptomatic BM, and 40% had symptomatic BM. In the course of the disease, asymptomatic BM was diagnosed more frequently (60.4%) than symptomatic BM (39.6%). Brain radiotherapy was received by 289 patients (23%), and 499 (39%) patients were also treated with targeted therapy after the diagnosis of their BM. Patient and tumor characteristics at the time of the diagnosis of advanced melanoma can be found in (Supplementary Table S1). Most patients (76.4%) were diagnosed with BM at the same moment they were diagnosed with their advanced melanoma.

### 3.2. Survival Tree

**Figure 1:**
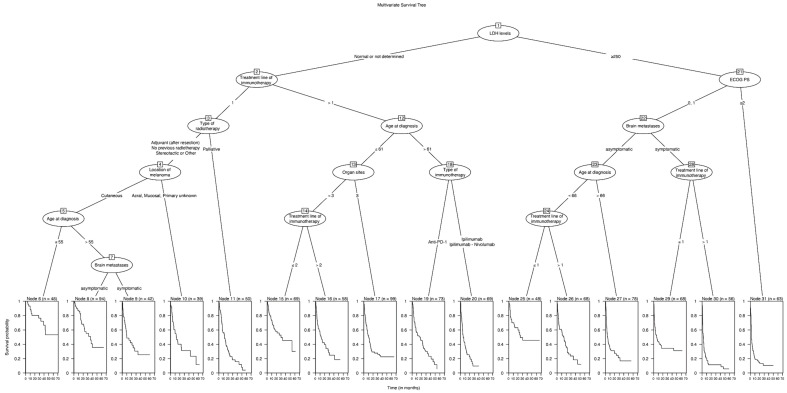
Multivariate survival tree of advanced melanoma patients with clinicopathological parameters as potential classifiers and overall survival as the response variable.

**Figure 2:**
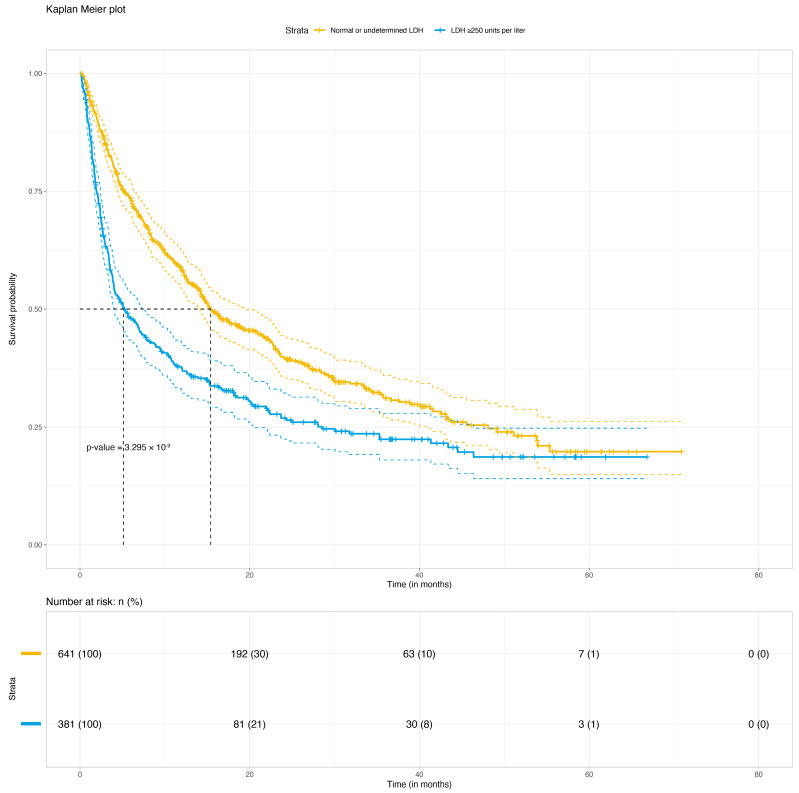
Kaplan–Meier curve of overall survival of advanced melanoma patients stratified by the strongest classifier: normal or undetermined LDH levels versus elevated LDH levels (>250 units per liter).

The median OS of the total population (node 1; *n* = 1022) was 12.2 months (95%CI 10.6–14.2) after the start of ICIs. The survival tree analysis resulted in 31 subgroups (Figure 1). The strongest classifier of OS in patients treated with ICIs after a diagnosis of BM was the serum LDH (normal or undetermined levels versus >250 units per liter). The median OS in patients with normal LDH levels (node 2; *n* = 641) was 15.4 months (95%CI 14.1–19.5), and in the elevated LDH groups (node 21; *n* = 381) it was 5.1 months (95%CI 4.0–7.3; *p* < 0.001) (Figure 2). In the elevated LDH group, the next prognostic covariate was the ECOG PS. Other covariates did not significantly classify OS in patients with an ECOG PS ≥2 (node 31; *n* = 63). In patients with an ECOG PS < 2 (*n* = 318), OS was most significantly influenced by the type of BM: asymptomatic or symptomatic. The most influential covariates in the group with asymptomatic BM were age and the treatment line in which the ICI treatment for BM was initiated. Age and type of given ICIs were not significant prognostic factors in the symptomatic BM group.

In patients with a normal LDH, the line of ICI treatment for BM was the most prognostic covariate. In patients diagnosed with BM in their first treatment line, the type of radiotherapy (adjuvant after resection, no radiotherapy, and stereotactic or other versus whole brain radiotherapy) given before the ICI treatment was the most important prognostic factor. In patients with adjuvant radiotherapy or no radiotherapy, significant classifiers were the type of melanoma (cutaneous versus acral, mucosal, or unknown primary) and age of diagnosis (≤55 or >55). The type of ICI (ipilimumab or ipilimumab–nivolumab versus anti-PD-1) was only significant in patients diagnosed with BM in a later treatment line and aged above 60 years. The median OS per node can be found in (Supplementary Table S2).

## 4. Discussion

This study focused on advanced melanoma patients with BM and the effect of ICI treatment on survival. The prognosis of the patients in our cohort was very heterogenous based on the different patient and disease characteristics. BM remain challenging to treat, and patients with BM are often excluded from clinical trials [12]. Using clinical parameters, this survival tree showed the different clinicopathologic characteristics associated with survival in advanced melanoma patients with asymptomatic or symptomatic BM.

Prior research has shown that the prognosis of patients with melanoma BM has improved in recent years. The median OS used to be between 4 and 5 months; however, it has improved to up to 14 months in more recent years [19,20,21]. This improvement is due to the advent of new systemic therapies such as immunotherapy and targeted therapy and the exploitation of new potential targets for treatment [22,23]. However, further improvements on the treatment of patients with melanoma BM are needed since the median OS remains poor.

The median OS of 12 months in our cohort is in line with the research by Bander et al., who described a median OS of 14 months in a comparable cohort of patients with melanoma BM [14]. The strongest clinical parameter identified in our study was the level of LDH and not, for example, the type of BM (asymptomatic or symptomatic). This finding aligns with earlier research investigating patients with or without BM treated with immuno- or targeted therapy [12]. LDH was already described as a useful marker at baseline and during treatment to predict the early response and progression in advanced melanoma patients treated with anti-PD-1 monotherapy [24]. Another study investigating biomarkers predicting the response to immunotherapy and OS found LDH as well as elevated baseline S100B to be associated with impaired OS [25]. Nosrati et al. [26] developed a clinical prediction scale to predict the response to anti-PD-1 monotherapy. They designed a 5-factor prediction scale, including elevated LDH, age < 65 years, female sex, a history of ipilimumab treatment, and the presence of liver metastases. Unfortunately, their cohort of 315 patients only included 50 patients with BM, making it difficult to apply the prediction scale to patients with melanoma BM. Starting treatment in a later treatment line had a negative impact on the prognosis, as reported by Derks et al. [27]. Interestingly, in our cohort, within patients with normal or undetermined LDH, melanoma subtype (cutaneous vs. non-cutaneous) was more influential on survival than the type of BM (symptomatic or asymptomatic). The type of ICI was only a significant classifier in patients with normal or undetermined LDH levels, diagnosed with BM in a later treatment line, and aged over 60 years. The fact that the choice of ICI (partly) depends on the patient’s LDH levels might explain why the type of ICI was not a strong classifier in our cohort. Now, ipilimumab–nivolumab is seen as the first choice of treatment for melanoma patients with BM based on the ABC trial. This multicenter open-label phase 2 trial included 79 patients and showed that a higher proportion of patients with melanoma BM respond to ipilimumab and nivolumab combination therapy, compared to ipilimumab or nivolumab monotherapy [16,28]. Other studies have also shown the added value of combination therapy when treating melanoma BM [29]. Ipilimumab–nivolumab was introduced as a treatment option for advanced melanoma in the Netherlands in 2016. Therefore, ipilimumab–nivolumab was not available for patients diagnosed before 2016, which might also contribute to the fact that the choice of ICI was not a strong classifier in our cohort.

Patients with symptomatic BM and a low LDH had a median survival of 10 months (95%CI 7.6–34.2), which was better than we expected based on the earlier literature [12]. Patients with elevated LDH levels and symptomatic BM in a later treatment line had a very poor median OS of 2.9 months (95%CI 1.7–4.4). When eligible, patients with elevated LDH levels and symptomatic BM might benefit more from other systemic therapies, such as targeted therapy or local treatment. Another treatment option could be switching to checkpoint inhibitors upon a response to targeted therapy [30].

This study does have limitations. First, data from a population-based registry were used as input for the survival tree. In these data, the choice of therapy for patients depended on the choice of the clinicians and the available knowledge, diagnostics, and treatments at that moment. This may lead to bias by indication. Second, due to the study’s retrospective nature, residual confounders might explain the observed associations. This warrants caution when interpreting the data. Furthermore, it is important to note that the median OS is not a sufficient discriminatory marker to assess the long-term survival of patients.

A strength of the current study is the large number of patients with BM included. The data in the DMTR are registered by independent, annually trained data managers. Data are registered in an online registry that warns data managers of inconsistent or missing values. To further ensure the data’s quality, the treating physicians check the registered data. Earlier studies have demonstrated the high quality of the DMTR [17].

Due to the increasing incidence of melanoma, increasing survival of advanced melanoma [31], and the higher number of patients with advanced disease who develop BM, [4,5,6,32] it is important to continue research for this vulnerable patient population, both in and outside the setting of clinical trials. The clinicopathological classifiers of this study can contribute to optimizing clinical studies in patients with BM and can aid doctors and their patients in giving an indication of the patients’ perspective based on their baseline and disease characteristics.

## 5. Conclusions

This study reported on clinicopathological parameters as classifiers of OS in patients with melanoma BM treated with ICIs. Patients with melanoma BM have a poor prognosis, and the median OS in our cohort ranged from 2.7 months to 35.7 months, depending on patient and tumor characteristics. LDH levels are the most important prognostic factor in this patient category. Researchers can use the prognostic classifiers identified in this present study, such as LDH, to optimize future clinical trials investigating melanoma patients with BM. In addition, clinicians can use this information to inform patients on their future perspective.

## Figures and Tables

**Table 1:** Patient and tumor characteristics. Patient and tumor characteristics of advanced melanoma patients at the time of diagnosis of brain metastases.

		All Patients (%)
N		1278
Median age [IQR]		61.0 [52.0, 70.0]
Sex	Male	802 (62.8)
	Female	476 (37.2)
ECOG PS	0	551 (43.1)
	1	502 (39.3)
	≥2	101 (7.9)
	Unknown	124 (9.7)
LDH levels (units/liter)	Undetermined	42 (3.2)
	Normal	770 (60.3)
	250–500	364 (28.5)
	500–750	61 (4.8)
	≥750	41 (3.2)
AJCC stage (8th edition)	IV-M1d	1278 (100.0)
Liver metastases	No	899 (70.3)
	Yes	362 (28.3)
	Unknown	17 (1.3)
Type of brain metastases	Yes, asymptomatic	772 (60.4)
	Yes, symptomatic	506 (39.6)
Organ sites	<3	429 (33.6)
	≥3	849 (66.4)
	Unknown	0 (0.0)
*BRAF* mutation status	*BRAF* mutant	768 (60.1)
	*BRAF* wildtype	461 (36.1)
	Undetermined	49 (3.8)
*NRAS* mutation status	*NRAS* mutant	258 (20.2)
	*NRAS* wildtype	768 (60.1)
	Undetermined	252 (19.7)
Type of immune checkpoint inhibitors for brain metastases	Ipilimumab	226 (17.7)
	Anti-PD-1	481 (37.6)
	Ipilimumab–nivolumab	571 (44.7)
Brain surgery prior to start of immune checkpoint inhibitors	No	1224 (95.8)
	Yes	54 (4.2)
Type of radiotherapy prior to start of immune checkpoint inhibitors	Adjuvant after resection	5 (0.4)
	Stereotactic	123 (9.6)
	Whole brain radiation therapy	151 (11.8)
	Other	10 (0.8)
	No radiotherapy	985 (77.1)
	Unknown	4 (0.3)
Anti-PD-1 therapy prior to diagnosis of brain metastases	No	1188 (93.0)
	Yes	90 (7.0)
Ipilimumab–nivolumab prior to diagnosis of brain metastases	No	1262 (98.7)
	Yes	16 (1.3)
Ipilimumab monotherapy prior to diagnosis of brain metastases	No	1222 (95.6)
	Yes	56 (4.4)
Targeted therapy prior to diagnosis of brain metastases	No	1150 (90.0)
	Yes	128 (10.0)
Targeted therapy after diagnosis of brain metastases	No	779 (60.9)
	Yes	499 (39.0)
Year of diagnosis of advanced melanoma	2013	96 (7.5)
	2014	108 (8.5)
	2015	173 (13.5)
	2016	151 (11.8)
	2017	201 (15.7)
	2018	226 (17.7)
	2019	203 (15.9)
	2020	120 (9.4)
Year of first treatment with ICI for brain metastases	2013	28 (2.2)
	2014	109 (8.5)
	2015	141 (11.0)
	2016	142 (11.1)
	2017	195 (15.3)
	2018	240 (18.80
	2019	241 (18.9)
	2020	174 (13.6)
	2021	8 (0.6)

## Data Availability

The data that support the findings of our study are available on request from the corresponding author.

## Supplementary Materials

The following supporting information can be downloaded at: https://www.mdpi.com/article/10.3390/cancers15112922/s1, Table S1: Patient and tumor characteristics; Table S2: Median Overall Survival; Supplementary Methods: Survival tree analysis.
