# Supplementary material for: A Survival Tree of Advanced Melanoma Patients with Brain Metastases Treated with Immune Checkpoint Inhibitors
Source: Cancers (Basel). 2023 May 26;15(11):2922. doi: 10.3390/cancers15112922 (PMC10252066; doi:10.3390/cancers15112922)
Supplement: Supplementary file 1 [file cancers-15-02922-s001.zip › cancers-2324666-supplementary.pdf]

SUPPLEMENTARY FILES

Supplementary Table S1 – Patient and tumor characteristics

Patient and tumor characteristics of patients with melanoma BM at the time of diagnosis of advanced melanoma.

		All patients (%)
**N**		1,278
**Median age [IQR]**		61.0 [52.0, 70.0]
**Sex**	Male	802 (62.8)
	Female	476 (37.2)
**ECOG PS**	0	593 (46.4)
	1	433 (33.9)
	≥2	125 (9.8)
	Unknown	127 (9.9)
**LDH levels**	Not determined	83 (6.5)
	Normal	741 (58.0)
	250-500	317 (24.8)
	500-750	67 (5.2)
	≥750	70 (5.5)
**AJCC stage (8th edition)**	IIIc unresectable	31 (2.4)
	IV-M1a	42 (3.3)
	IV-M1b	60 (4.7)
	IV-M1c	165 (12.9)
	IV-M1d	977 (76.4)
	Unknown	3 (0.2)
**Brain metastases**	No	291 (22.8)
	Yes, asymptomatic	472 (36.9)
	Yes, symptomatic	505 (39.5)
	Unknown	10 (0.8)

**Liver metastases**	No	934 (73.1)
	Yes	332 (26.0)
	Unknown	12 (0.9)
**Organ sites**	<3	534 (41.8)
	≥3	739 (57.8)
	Unknown	5 (0.4)
**Mutation status**	*BRAF*	768 (60.1)
	NRAS	258 (20.2)
**Year of diagnosis of advanced melanoma**	2013	96 (7.5)
	2014	108 (8.5)
	2015	173 (13.5)
	2016	151 (11.8)
	2017	201 (15.7)
	2018	226 (17.7)
	2019	203 (15.9)
	2020	120 (9.4)

Supplementary Table S2 – Median Overall Survival

Median overall survival by treatment node.

Treatment Node number	Number of patients	Median Overall Survival	Lower 95% CI	Upper 95% CI
1	1,022	12.2	10.6	14.2
2	641	15.4	14.1	19.5
3	273	23.1	17.4	33.2
4	223	31.8	23.0	42.5
5	184	35.7	26.3	Not Reached
6	48	Not Reached	42.5	Not Reached
7	136	26.3	21.3	40.1
8	94	35.7	23.1	Not Reached
9	42	10.0	7.6	34.2
10	39	12.6	7.2	Not Reached
11	50	12.5	7.6	18.9
12	368	12.0	10.2	14.8
13	226	13.9	11.0	17.6
14	127	21.4	12.7	30.0
15	69	30.0	16.1	Not Reached
16	58	12.2	7.4	27.4
17	99	11.0	7.5	14.1
18	142	9.3	6.8	14.2
19	73	17.8	8.5	24.9
20	69	6.1	4.4	11.5
21	381	5.1	4.0	7.3
22	318	6.7	4.6	10.7
23	194	11.0	6.3	18.2
24	116	18.2	12.0	24.3
25	48	25.0	19.9	Not Reached
26	68	14.8	9.7	21.9
27	78	4.7	3.5	9.0
28	124	3.9	2.9	6.7
29	68	6.8	3.4	16.8
30	56	2.9	1.7	4.4
31	63	2.7	2.4	4.7

SUPPLEMENTARY METHODS

Survival tree analysis

Survival tree analysis is used to classify samples with clinicopathological information based on the difference between survival probabilities at certain time intervals. All clinicopathologic parameters were used as classifiers.

Survival tree analysis was performed using the following steps:

1. For each of the classifiers (C_i):
   a. If the classifier is a numeric variable,
      i. For each possible weight of the classifier as a cut-off (c):
         1. Obtain two subsets of the samples.
            a. Samples having weights < cut-off weight
            b. Or samples having weights >= cut-off weight
         2. Obtain survival probabilities for both the subsets.
         3. Compare the survival probabilities using logrank test and obtain logrank statistic ($LRC_{i,c}$).
      ii. Obtain the optimum cut-off c (oc_i) for which logrank statistic $LRC_{i,c}$ is maximum. Assign maximum of $LRC_{i,c}$ as max_LRC_i.
   b. If the classifier is a categorical variable,
      i. For each possible combination of different levels of the classifier:
         1. Obtain two subsets of the samples A and B.
         2. Obtain survival probabilities for both the subsets.
         3. Compare the survival probabilities using logrank test and obtain logrank statistic ($LRC_{i,AB}$).

ii. Obtain the optimum combination of levels in two subsets A, B (oc_i) for which logrank statistic $LRC_{i,AB}$ is maximum. Assign maximum of $LRC_{i,AB}$ as max_LRC_i.

2. Obtain the most significant classifier along with the optimum cut-off/combination of levels (oc) for which max_LRC_i is maximum among all classifiers.
3. Classify the samples into two subsets (subset_1 and subset_2) using the most significant classifier and optimum cut-off/combination of levels.
4. In each of the subsets of the dataset, repeat steps 1,2 & 3 till the following constraints are maintained.
    a. Number of samples in subset_1 + number of samples in subset_2 >= 100
    b. number of uncensored events in subset_1 + number of uncensored events in subset_2 >= 50
    c. number of samples in subset_1 or number of samples in subset_2 >= 34

Thereafter, 10,000 iterations of the above 5 steps were performed using random 80% of the samples each time to investigate robustness of the significance of the classifiers obtained from survival tree analysis mentioned above.

To assess the goodness-of-fit of the survival tree, the following steps were conducted:

1. A new variable called survival cohorts was created to store the terminal node number in the survival tree of each sample.
2. Univariate Cox regression with this new variable survival cohorts as a predictor was performed to assess its association with OS.
3. Concordance statistic for this Cox regression model was obtained to evaluate the classification power of the survival tree.

To quantify the robustness of the survival tree, the following steps were conducted:

1. Survival tree analysis was conducted 10,000 times using random 80% of the samples each time.

2. For the survival tree with all samples
    a. For each classifier C
        i. $NOS\_node_{C,n}$ was obtained as the number of samples present in node n where C was the most significant classifier.
        ii. $Max\_NOS\_node_{C}$ was obtained as maximum of $NOS\_node_{C,n}$.
        iii. $R\_all\_samples\_survival\_tree_{C}$ was obtained as rank of $Max\_NOS\_node_{C}$.
3. For each of the 10,000 iterations *i*
    a. For each classifier *C*
        i. $NOS\_node_{C,n}$ was obtained as the number of samples present in node *n* where *C* was the most significant classifier.
        ii. $Max\_NOS\_node_{C}$ was obtained as maximum of $NOS\_node_{C,n}$.
    b. $Robustness\_statistic_{i}$ was obtained as the Pearson correlation coefficient between $Max\_NOS\_node_{C,i}$ and $Max\_NOS\_node_{C}$

The robustness of the survival tree was presented by the median of $Robustness\_statistic_{i}$.
